# Identification of spastic ataxia-related proteins via comparative proteomic analysis of the cerebellum of conditional *Ankfy1* knockout mice

**DOI:** 10.1038/s41598-025-06398-8

**Published:** 2025-07-01

**Authors:** Rong Fu, Man Ding, Tong Yin, Linlin Zheng, Yue Liu, Hang Yu, Rumeng Zhou, Zuneng Lu

**Affiliations:** Department of Neurology, Renmin Hospital of Wuhan University, Wuhan, 430060 Hu Bei Province China

**Keywords:** Ankfy1, Purkinje cell, Spastic ataxia, Data-independent acquisition, Genetics, Neuroscience, Neurology

## Abstract

Ankyrin repeat and FYVE domain containing 1 (ANKFY1) is an indispensable protein in the development of cerebellar Purkinje cells. Our preliminary study revealed that its absence caused progressive spastic ataxia, accompanied by the loss of Purkinje cells in mice. Here, we generated *Ankfy1*-floxed (*Ankfy1*^f/f^) mice, in which conditional inactivation of the *Ankfy1* gene was achieved specifically in cerebellar Purkinje cells via crossing with a transgenic mouse strain expressing Cre recombinase under the regulatory control of the Purkinje cell protein 2 (PCP2) promoter. We employed data-independent acquisition (DIA) mass spectrometry to compare the protein expression profiles in cerebellar samples. The samples were obtained from two groups of male mice. The first group consisted of three Pcp2-Cre; *Ankfy1*^f/f^ male mice, where the *Ankfy1* gene was conditionally knocked out in Purkinje cells, and these mice were designated as the conditional knockout (CKO) group. The second group included three Cre-negative; *Ankfy1*^f/f^ littermate male mice served as the wild-type (WT) control group. The results identified 69 (45 upregulated and 24 downregulated) differentially expressed proteins (DEPs) in CKO vs. WT male mice with a 1.5-fold change. Enrichment analyses of these DEPs based on the Gene Ontology (GO), Orthologous Groups of Proteins (COG), and Kyoto Encyclopedia of Genes and Genomes (KEGG) databases revealed functional clusters associated with neuronal cell morphogenesis, extracellular structures, regulation of Rho-GTPase, calcium signaling pathway, etc. *Itgb2*, which was upregulated in CKO mice, was the top hub gene according to protein–protein interaction (PPI) analysis. We selected seven interesting differentially expressed genes (*Arhgdib*, *Impa2*, *Pcp2*, *Pcp4*, *Ppp1r17*, *Rhobtb2*, and *Cdc123*) for further validation. *Arhgdib* and *Imp2a* expression was increased in the cerebellum of CKO male and female mice, and *Pcp2* and *Pcp4* expression was decreased. Western blotting and immunofluorescence verified the upregulation of ARHGDIB and the downregulation of PCP2 in the cerebellum. Our proteomic analysis of conditional *Ankfy1* knockout mice may guide future research to help develop treatments for progressive spastic ataxia.

**Supplementary Information:**

The online version contains supplementary material available at 10.1038/s41598-025-06398-8.

## Introduction

Spastic ataxia is a syndrome characterized by the combination of cerebellar ataxia with spasticity and pyramidal features. The causes of this syndrome can be divided into acquired and genetic disorders^1,2^. Acquired spastic ataxias may arise from structural or vascular pontocerebellar abnormalities, inflammatory central nervous system (CNS) diseases, multiple sclerosis, CNS infections, and so on^1^. There are 82 subtypes of hereditary spastic paraplegia, such as Friedreich ataxia, autosomal recessive ataxia of Charlevoix-Saguenay (ARSACS), and hereditary spastic paraplegia type 7, which have been described thus far^2^.

ANKFY1 is a 130-kDa cytoplasmic protein that was first identified and named Ankhzn (Ankyrin repeats hooked to a zinc finger motif) by Kazuhisa Ito et al.^3^. Its structure is unique, with the N-terminus containing a BTB/POZ motif, 17 ARs in the middle, and the C-terminal region containing a double zinc finger motif^4^. ANKFY1 is a PI (3)P-binding Rab5 effector via its FYVE domain that localizes to early endosomes and promotes their formation, which plays an active role in the generation of macropinosomes^5,6^. Researchers have verified its role in maintaning cerebellar Purkinje cells^7^. In 2007, Bouslam et al. first reported that it may be involved in autosomal recessive spastic ataxia^8^. Our previous study demonstrated that ankfy1 knockout mice develop ARSACS with Purkinje cell loss in the mouse cere^9,10^, but the underlying mechanisms remain unclear.

This study aimed to explore the pathogenesis of abnormal ANKFY1-mediated spastic ataxia more precisely in the cerebellum through DIA-based proteomics and bioinformatics analyses. A mouse model of conditional *Ankfy1* knockout in cerebellar Purkinje cells was established. Mass spectrometry was used to investigate DEPs in the cerebellum between WT mice and conditional *Ankfy1* knockout mice. We also addressed the potential implications of our findings on the pathogenesis of ANKFY1 in recessive spastic ataxia, such as glial cell activation, changes in the collagen ligand integrin, and the regulation of Rho-GTPase and PCP2-mediated loss of Purkinje cells.

## Materials and methods

### Experimental animals

All animal experiments were approved by the Animal Care and Use Committee of Renmin Hospital of Wuhan University (ethical approval number: 20200613). *Ankfy1*-floxed (*Ankfy1*^f/f^) male mice and Pcp2-Cre female mice with a C57BL/6J genetic background (backcrossed for at least 10 generations, N10) were obtained from GemPharmatech Co., Ltd. (Nanjing, China). To generate conditional knockout mice, *Ankfy1*^f/f^ mice were crossed with Pcp2-Cre mice to produce Pcp2-Cre*; Ankfy1*^f/w^ heterozygous offspring (F1 generation). These F1 mice were then intercrossed under specific pathogen-free conditions at the Center Animal Laboratory of Renmin Hospital of Wuhan University to obtain F2 generation mice for experiments. This study used Pcp2-Cre; *Ankfy1*^f/f^ mice as the CKO group, while Cre-negative littermate *Ankfy1*^f/f^ mice were used as WT controls. The deletion of the *Ankfy1* gene in the transgenic mice was validated by polymerase chain reaction (PCR). The Cre genotyping reaction yielded a 567-bp band using the following primers: forward primer (5’-ATTCTCGTGGAACTGGATGG-3’) and reverse primer (5’-GGACAGGTAATGGTTGTCTGG-3’). The following primers were used to separate wild-type, heterozygous, and homozygous flox sequences: forward primer (5’-CTGCCTACACTTCTAGGCCTGGTTT-3’) and reverse primer (5’-GGATTTTAAGTGCCTGGCATCGG-3’). By PCR, a single 309-bp WT band was obtained for the WT, a 410-bp single targeted band was obtained for the homozygote, and a band and a targeted band were obtained for the heterozygote. All methods were performed under the ARRIVE guidelines and relevant guidelines and regulations.

### Mass spectrometry

#### Protein extraction and digestion

Six-month-old male mice were chosen for mass spectrometry because CKO mice had poorer rotarod performance at that age than WT mice. In order to minimize biological variation due to sex differences, including hormonal cycles or metabolic traits, male mice were used in this study. Six-month-old male WT and CKO mice (n = 3 for each group) were deeply anesthetized with an intraperitoneal injection of pentobarbital sodium in phosphate-buffered saline (PBS) solution at 40 mg/kg. Their hearts were exposed, and a PBS solution-aspirated needle was inserted into the left ventricle; the right ear was severed, and PBS solution was gradually infused until the livers were bloodless. Then, the spinal cord and the cerebellar tissues were isolated. The cerebellar tissues were subjected to lysis by adding a lysis buffer consisting of 1.5% sodium dodecyl sulfate (SDS) and 100 mM Tris–Cl. After thorough mixing, the samples underwent tissue homogenization and centrifugation, and the supernatants were collected. Subsequently, the proteins in the supernatant were precipitated using the acetone precipitation method.

The resulting protein pellets were resuspended in a buffer containing 8 M urea and 100 mM Tris–Cl. Dithiothreitol was then added to the resuspended samples, which were incubated at 37 °C for 1 h to reduce disulfide bonds. Afterward, iodoacetamide (IAA) was introduced, and the alkylation reaction was conducted at room temperature in the dark to modify sulfhydryl groups covalently.

Protein concentration was quantified using the Bradford assay. In preparation for enzymatic digestion, 100 mM Tris–HCl buffer was added to the reduced and alkylated samples, diluting the urea concentration to below 2 M. Trypsin was added at an enzyme-to-protein mass ratio of 1:50, and the samples were incubated with shaking at 37 °C overnight to facilitate proteolytic digestion. The following day, the digestion reaction was terminated by adding trifluoroacetic acid. The supernatants were then collected and subjected to desalting using Sep-Pak C18 cartridges. After vacuum-drying, the samples were stored at -20 °C until further analysis.

#### DIA analysis

The mass spectrometry data were acquired using a liquid chromatography-mass spectrometry system that coupled an Orbitrap Exploris 480 mass spectrometer with an EASY-nLC 1200 liquid chromatography. The peptide samples were dissolved in the loading buffer, aspirated by the autosampler, and then bound to an analytical column (75 μm * 25 cm, C18, 1.9 μm particle size, 100 Å pore size) for separation. An analytical gradient was established using two mobile phases: mobile phase A, which consisted of 0.1% formic acid, and mobile phase B, which was composed of 0.1% formic acid and 80% acetonitrile. The flow rate of the liquid chromatography was set at 300 nL/min.

The mass spectrometry data were collected in the DIA mode. Each scan cycle included one MS1 scan (resolution, R = 60,000; automatic gain control target value, AGC = 3 × 10⁶; maximum injection time, Max IT = 30 ms; scan range = 350–1250 m/z) and forty MS2 scans with variable windows (resolution, R = 30,000; automatic gain control target value, AGC = 1000%; maximum injection time, Max IT = 50 ms). The FAIMS (High-Field Asymmetric Waveform Ion Mobility Spectrometry) function was enabled with a compensation voltage (CV) of -45, and the collision energy was set at 30.

#### Data analysis

The raw DIA data files were analyzed using the DIA-NN software (v 1.8). The database used for retrieval was the proteomic reference database of mice from Uniprot (dated August 26, 2020, containing 17,053 protein sequences). A spectral library was predicted through the deep learning algorithm in DIA-NN. The raw DIA data were extracted using the predicted spectral library and the spectral library obtained from the MBR (Match Between Runs) function to acquire protein quantification information. The final results were filtered at the precursor ion and protein levels with a 1% false discovery rate (FDR). The filtered proteomic quantification information was used for subsequent analyses.

Subsequently, functional enrichment analysis using the hypergeometric test identified significantly enriched functional categories of DEPs, with a P-value < 0.05, indicating significance and biological relevance. Relevant databases were used for bioinformatic analysis of DEPs. The evolutionary genealogy of Genes: Nonsupervised Orthologous Groups (EggNOG) v5.0 database was used to annotate COG clusters. The UniProt database was used to annotate the GO of the proteome, and DEPs were classified based on three categories of GO annotations: biological processes (BP), cellular components (CC), and molecular functions (MF). The KEGG database was used for pathway enrichment analysis^11–13^. PPI analysis was conducted using the String database.

### Validation of differential proteins

#### Real-time quantitative PCR (RT‒qPCR)

Seven-month-old mice that presented with Purkinje cell loss were used for further validation. Cerebellar samples, including six males and six females in the WT group and six males and six females in the CKO group, were collected and used to extract total RNA according to the TRIzol reagent protocol (Vazyme, China). According to the manufacturer’s guidelines, 1 μg of RNA from each sample was converted to cDNA with a cDNA synthesis kit (R323-01, Vazyme, China). Then, we used RT‒qPCR (G3320, Servicebio, China) to analyze the expression levels of downregulated genes, including *Pcp2*, *Pcp4*, *Ppp1r17*, and *Cdc123*, and upregulated genes, including *Arhgdib*, *Impa2,* and *Rhobtb2*. The relevant primers used were as follows: mouse *Pcp2* forward primer (5'-ATGGACGACCAGCGTGTAAC-3’), reverse primer (5'-CCTTGGGGCCG ATAGGTTG-3’); mouse *Pcp4* forward primer (5’- CAGGCGTTCAGACAGGTGAA-3’), reverse primer (5'-CGGCACTTTGTCTCTCACTCA-3’); mouse *Ppp1r17* forward primer (5'-GGAGCGACCAACGGAAAAGA-3’), reverse primer (5’- CACTAGGACT GTGATCCTGCC-3’); mouse *Cdc123* forward primer (5’- GCATGTTAGTCACT GTCAGTTCT-3’), reverse primer (5’- CTCCCAGCAGTGTTTTCTTGTA-3’); mouse *Impa2* forward primer (5’- AGAGGGAGAGTTGGTGCAG-3’), reverse primer (5’- GTTTCTGTCACAAGATCGGCA-3’); mouse *Arhgdib* forward primer (5’- ATGAC GGAGAAGGATGCACAG-3’), reverse primer (5’- CTCCCAGCAGTGTTTTCTT GTA-3’); mouse *Rhobtb2* forward primer (5’- GACCGGCGTTTTGCTTATGG-3’), reverse primer.

#### Western blotting

Total protein was extracted quickly from the cerebellum samples of six males and six females in the WT group and five males and six females in the CKO group. After each sample was added to a protease inhibitor-containing radioimmunoprecipitation (RIPA) buffer, the samples were chilled for 15 min. After 15 s of sonication, the samples were centrifuged at 4 °C for 15 min at 12,000 rpm, after which the supernatant was collected. Protein quantification was performed using the Bradford technique (Bio-Rad). Subsequently, each supernatant was homogenized with RIPA buffer and added to SDS–polyacrylamide gel electrophoresis sample loading buffer before being placed in a 100 °C metal bath for 5 min. Homogenates were separated on an SDS–polyacrylamide gel and transferred onto a polyvinylidene fluoride membrane (Millipore). The membranes were blocked in 5% nonfat milk for one hour and incubated with specific antibodies overnight at 4 °C. Immunoblots were probed with mouse polyclonal glyceraldehyde 3-phosphate dehydrogenase (GAPDH) (60004-1-lg, Proteintech, China, 1:100,000), mouse polyclonal anti-PCP2 (sc-137064, Santa Cruz, 1:300), and monoclonal anti-ARHGDIB (sc-376473, Santa Cruz, 1:200) antibodies. The membrane was incubated for 1 h at room temperature with horseradish peroxidase-conjugated anti-mouse secondary antibodies (BL001A, Biosharp, China, 1:10,000). Western blot enhanced chemiluminescence substrate (Bio-Rad Laboratories, Inc.) was used to detect reactive bands as directed by the manufacturer. We used GAPDH for normalization. Densitometric analyses of the Western blot bands were performed with ImageLab 6.0 software (Bio-Rad Laboratories, Inc.) and with open-source ImageJ software.

#### Immunostaining

Immunofluorescence staining was performed on six seven-month-old male mice from three WT and three CKO groups. After the mice were anesthetized, the hearts of the mice were perfused with 4% paraformaldehyde, and the brain tissues were dissected and placed in 4% paraformaldehyde at 4 °C for 24 h before being dehydrated in 30% sucrose solution until the tissues sank to the bottom of the bottle. Then, the brain tissues were removed, added to the optimal cutting temperature compound, and placed in liquid nitrogen for 1 min. Sagittal sections were generated using a freezing microtome with a thickness of 30 μm and stored at -20 °C.

Frozen sections were removed from -20 °C, thawed at room temperature for 30 min, painted in a group around the tissue, and then washed in PBS three times for five minutes each. They were then blocked with 5% bovine serum albumin (BSA)/PBS at room temperature for 30 min, followed by overnight incubation at 4 °C with mouse polyclonal anti-PCP2 (sc-137064, Santa Cruz, 1:100) and monoclonal anti-ARHGDIB (sc-376473, Santa Cruz, 1:50) antibodies. The slides were incubated with a fluorescent secondary antibody for one hour at 37 °C. 4',6-Diamidino-2-phenylindole was added for ten minutes before the cells were washed three times with PBS for 10 min each time.

Consecutive sagittal sections were observed using an orthomosaic microscope (Olympus fully automated fluorescence microscope, BX63, Japan) through a 20 × objective lens. The average fluorescence intensity of PCP2 in the molecular layer and Purkinje cell layer of the cerebellum, as well as the average fluorescence intensity of ARHGDIB in the entire cerebellar layer, were statistically analyzed using ImageJ software.

#### Statistical analysis

Statistical differences were analyzed using GraphPad Prism 8.0 software. Normally distributed data are expressed as the mean ± standard deviation $\left( {\overline{\chi } \pm s} \right)$, and the *t*-test for independent sample means was used to compare the two groups. Differences were considered statistically significant at P < 0.05. We used two-way ANOVA followed by Bonferroni’s multiple comparisons test in GraphPad Prism 8.0 software to analyze the RT‒qPCR data.

## Results

### Identification of mouse genotypes

**Fig. 1:**
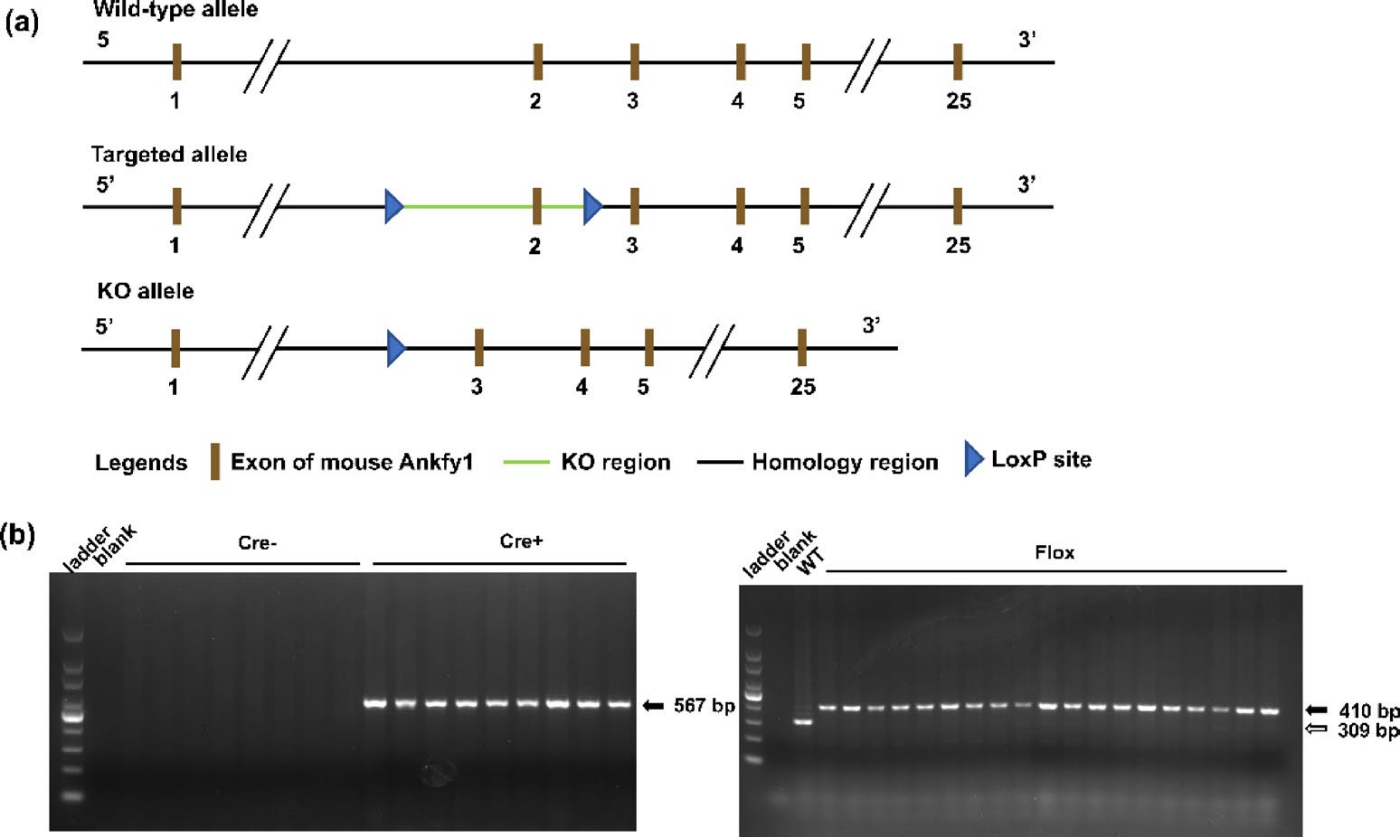
Generation of WT and CKO mice. (**a**) Schematic representation of the targeting constructs. The flox sequence was inserted into exon 2 of the ANKFY1 locus. (**b**) PCR results. Cre+ presented a 567-bp band. The homozygous flox produced a 410-bp single targeted band, whereas the WT produced a 309-bp band.

**Fig. 2:**
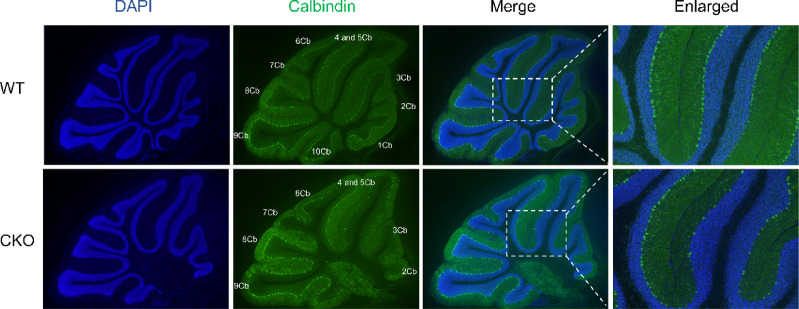
Loss of cerebellar Purkinje cells induced by conditional knockout of *Anky1*. 1st cerebellar lobule = 1Cb, 4th and 5th cerebellar lobules = 4 and 5Cb, 5th cerebellar lobule = 5Cb, 6th cerebellar lobule = 6Cb, 7th cerebellar lobule = 7Cb, 8th cerebellar lobule = 8Cb, 9th cerebellar lobule = 9Cb, and 10th cerebellar lobule = 10Cb. Scale bars: 200 µm (merge, for magnification at 40×) and 500 µm (enlarged, for magnification of 200×).

An *Ankfy1*-floxed (*Ankfy1*^f/f^) transgenic mouse model was generated in which the LoxP sites were located on exon 2 of the *Ankfy1* gene (Fig. 1a). Then, the *Ankfy1*^f/f^ mice were crossed with Pcp2-Cre mice, and finally, Pcp2-Cre; *Ankfy1*^f/f^ mice were obtained. Agarose gel electrophoresis revealed that the genotypes of the WT, *no-Cre, Ankfy1*^f/f^ and CKO groups were Pcp2-Cre; *Ankfy1*^f/f^, indicating that the conditional Ankfy1 knockout mouse model of cerebellar Purkinje cells was successfully generated (Fig. 1b). In this study, we first observed differences in gait between the WT and CKO groups at six months of age; Purkinje cell loss occurred in seven-month-old CKO mice (data not shown). Conditional *Ankfy1* knockdown in 1-year-old male mice resulted in Purkinje cell loss throughout the mouse cerebellum, with the most significant loss occurring in lobules 3–5 (Fig. 2).

### Quantitative analysis of 59 DEPs

**Fig. 3:**
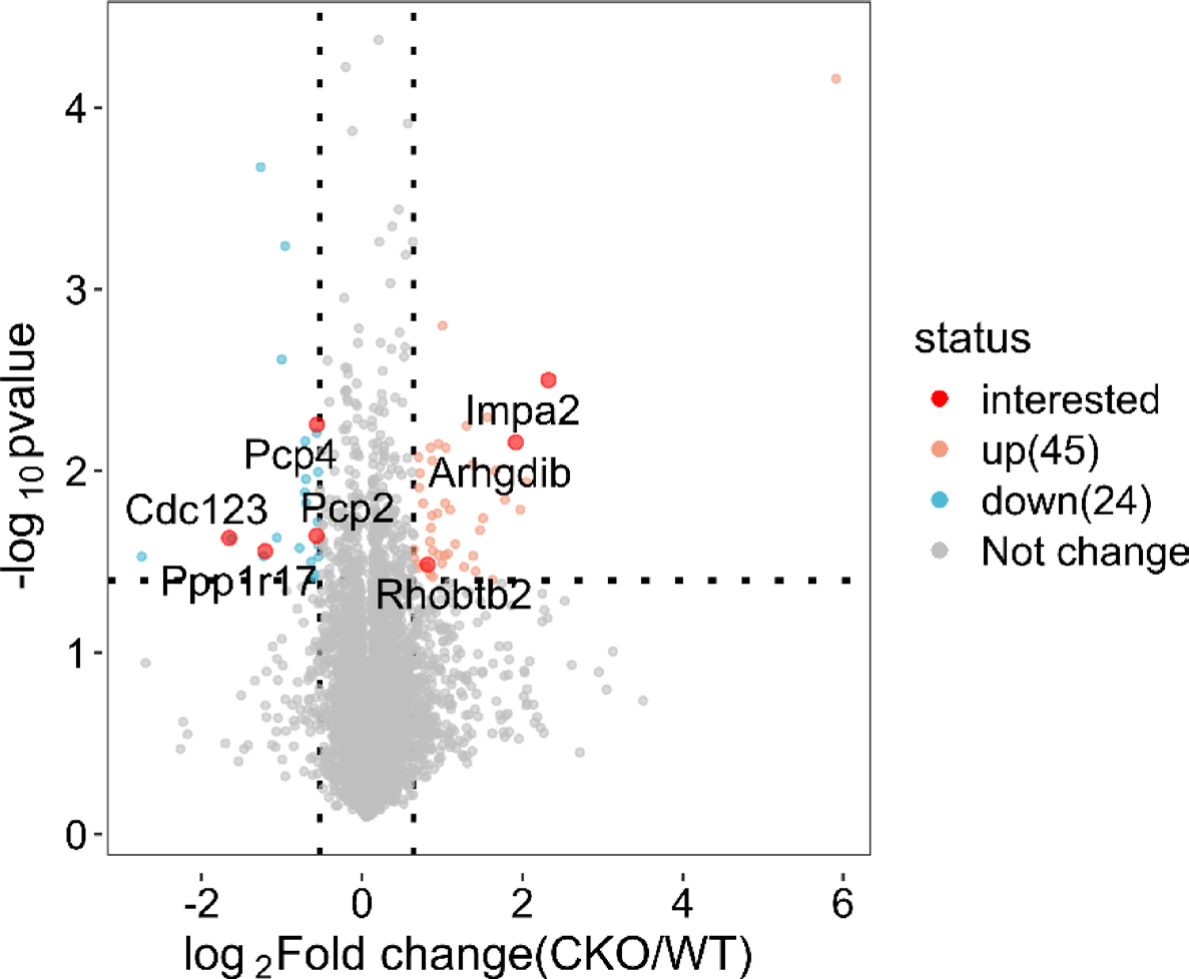
Volcano map of DEPs. The x-axis represents log2-transformed fold changes in protein expression, while the y-axis indicates the corresponding -log10-transformed P-values. Orange dots denote proteins with significant changes, blue dots signify considerably downregulated proteins, gray dots represent proteins with no significant alterations, and proteins of interest are highlighted with red dots.

**Fig. 4:**
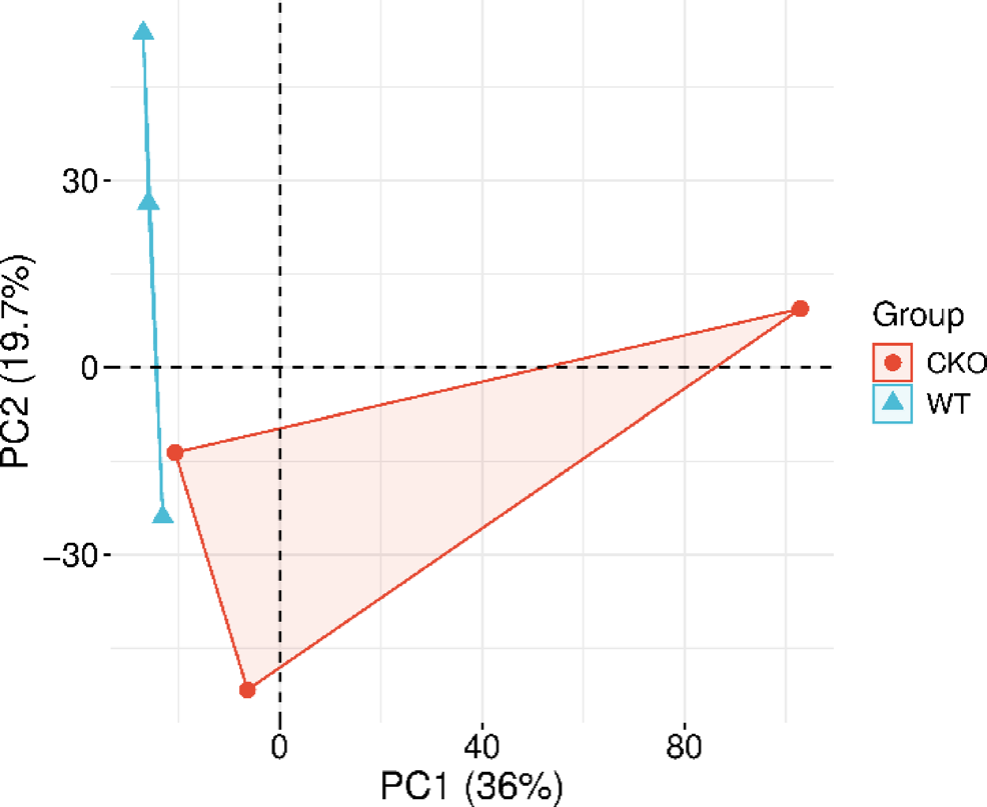
Principal component analysis (PCA) of DEPs between the CKO and WT groups.

**Fig. 5:**
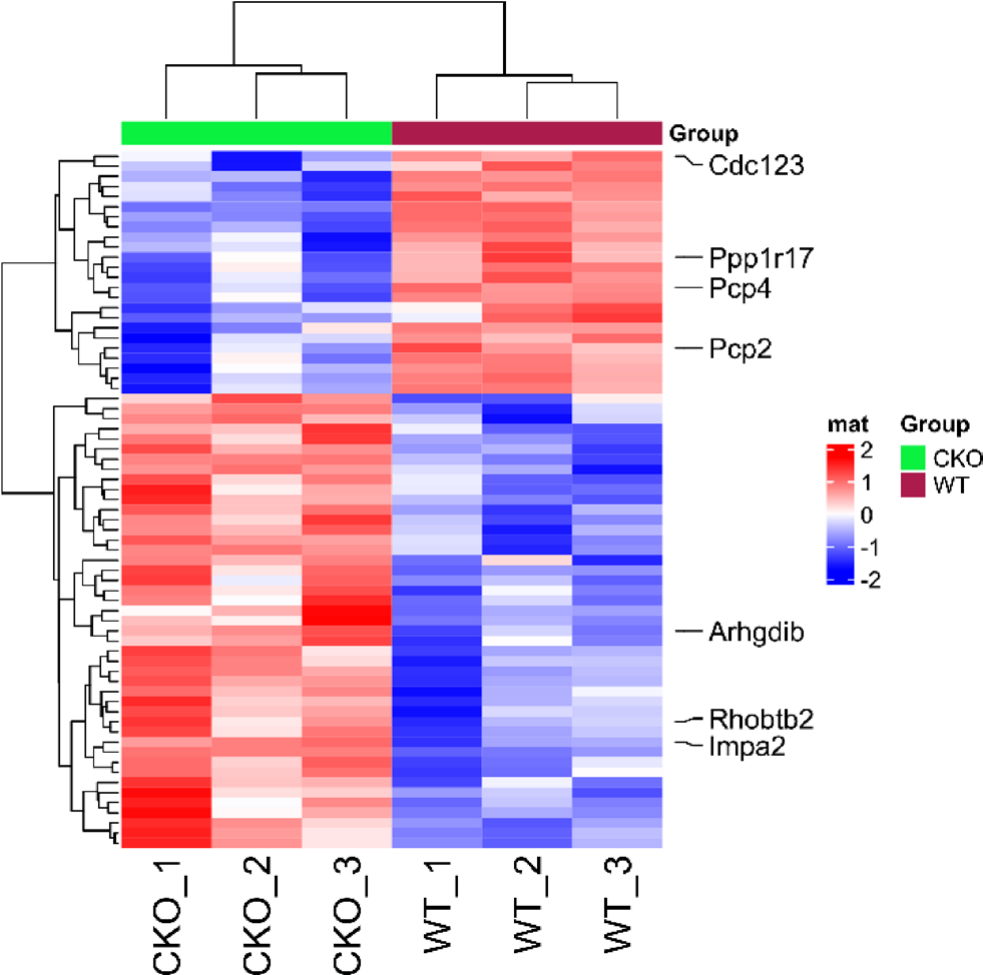
Heatmap of the abundance levels of DEPs in CKO vs. WT. Each row represents a protein, and each column represents a sample group, in which a higher red represents greater upregulation and a higher blue indicates greater downregulation.

DIA mass spectrometry was used to establish and analyze a spectral library of mouse cerebellar proteins obtained from three WT mice and three CKO mice. A total of 58,711 peptides and 7224 proteins were identified. The screening criteria for significant DEPs were a fold change ≥ 1.5 and P < 0.05. Compared with those in the WT group, 69 proteins were differentially expressed in the CKO group. Among these DEPs, 45 were upregulated, and 24 were downregulated (Fig. 3). Principal component analysis (PCA) of DEPs demonstrated apparent biological repeatability within the same group and significant variability between these two groups, suggesting that we can clearly distinguish the CKO group from the WT group (Fig. 4). These DEPs were then used to create a heatmap, which intuitively depicted expression variations between the different groups (Fig. 5). Function and UniProt information for the upregulated and downregulated DEPs are provided in Supplementary Tables 1 and 2, respectively.

### GO enrichment analysis revealed that conditional *Ankfy1* knockout predominantly causes alterations in response to stimulus-related proteins

**Fig. 6:**
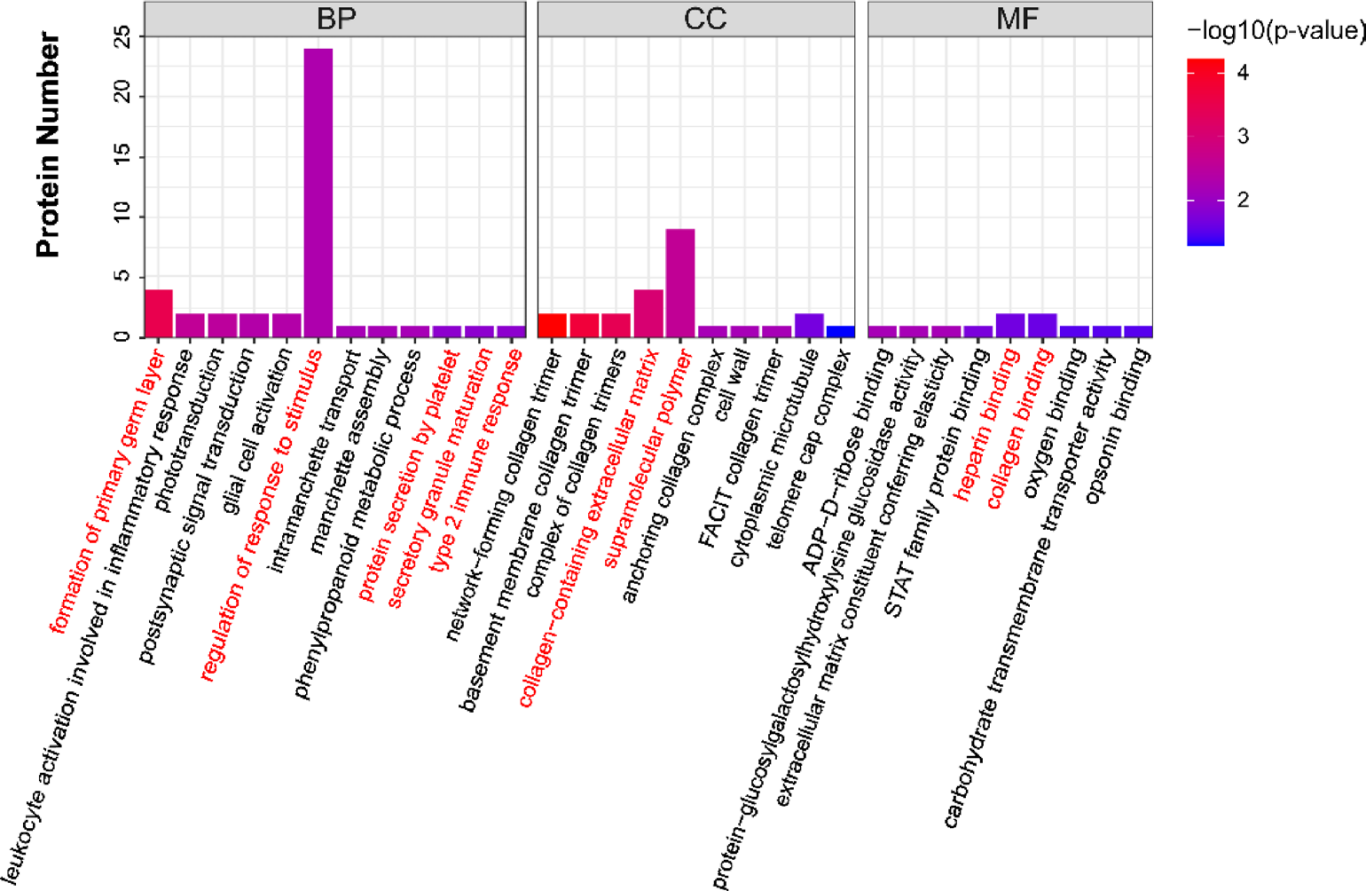
GO enrichment of the top 10 DEPs between the CKO and WT groups. Terms referenced in the text are marked in red.

The GO database is a classification system used to represent various properties of genes and gene products. It is divided into three major categories: BP, CC, and MF. Among the 69 differentially expressed proteins, 63 had GO annotations. Based on statistical significance (p-value), the top ten entries from each functional category were selected to construct the graph (Fig. 6), focusing on the most prominent enrichments. The BP category ultimately contains 12 items. “Regulating the response to a stimulus” is a representative GO term, followed by the “formation of the primary germ layer”. As “type 2 immune response”, “secretory granule maturation”, and “protein secretion by platelet” have the same p-values and jointly hold the tenth rank. The CC terms “supramolecular polymer” and “collagen-containing extracellular matrix” are highly representative. Among the MF, “heparin binding” and “collagen binding” are highly representative (Fig. 6). The proteins corresponding to each term are presented in Supplementary Table 3.

### COG classification indicates that ANKFY1 may be involved in extracellular structures

**Fig. 7:**
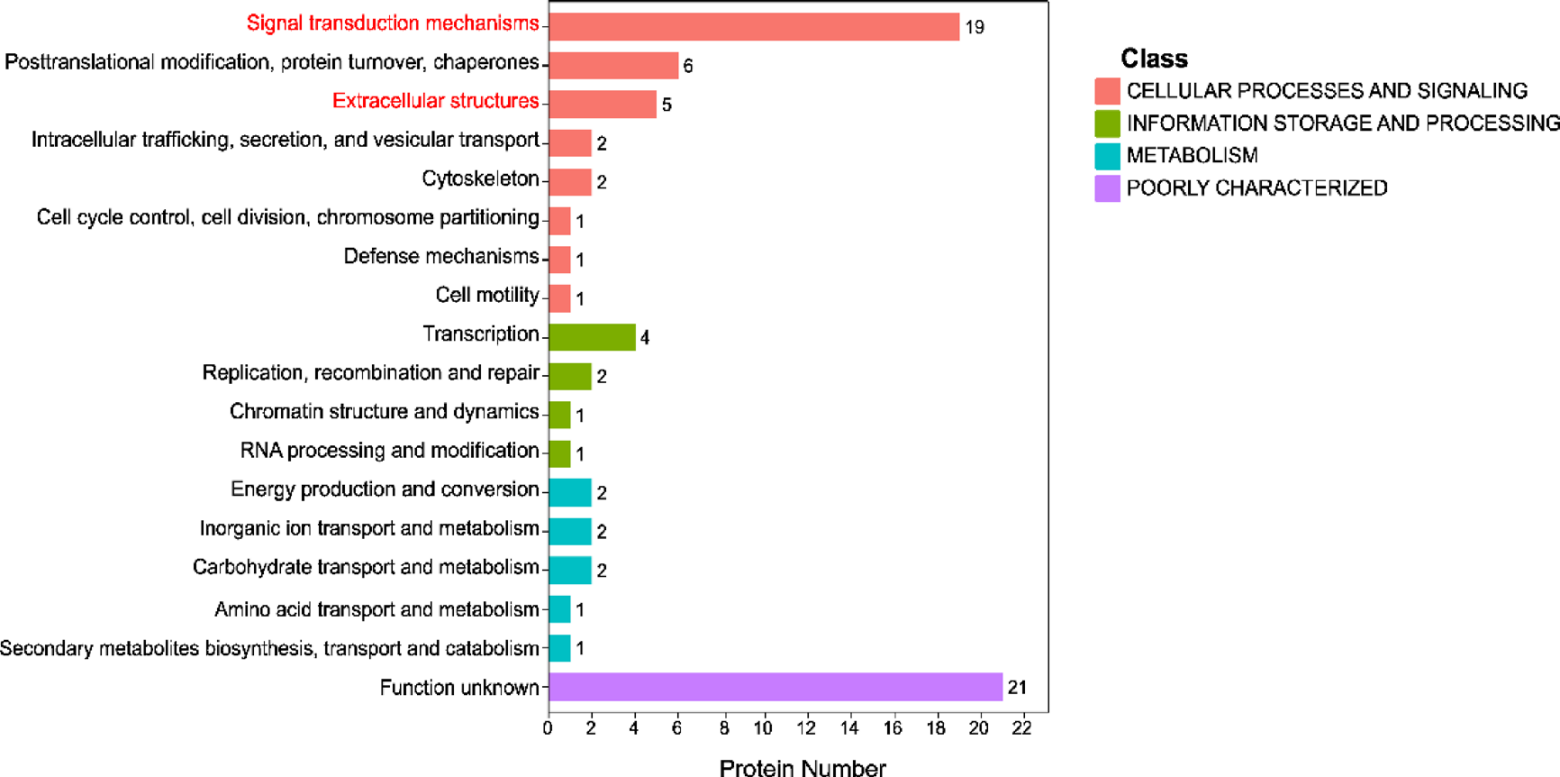
COG classification of DEPs. Terms referenced in the text are marked in red.

We conducted COG classification of the DEPs via the EggNOG v5.0 database (Fig. 7). This result demonstrated that 21 DEPs were poorly characterized in COG classification information. Among all the categories, only the entry of “extracellular structures” showed statistical significance with* p* < 0.05, and five differentially expressed proteins were enriched in this entry. The entry with the highest number of differentially expressed proteins enriched was "signal transduction mechanisms," which showed a predominant association of the DEPs with signal transduction mechanisms. Unfortunately, it did not exhibit statistical significance at* p* > 0.05. Proteins in each term can be displayed in the Supplementary Table 4.

### KEGG pathway analysis revealed that ANKFY1 might be involved in adhesion, protein digestion, and absorption

**Fig. 8:**
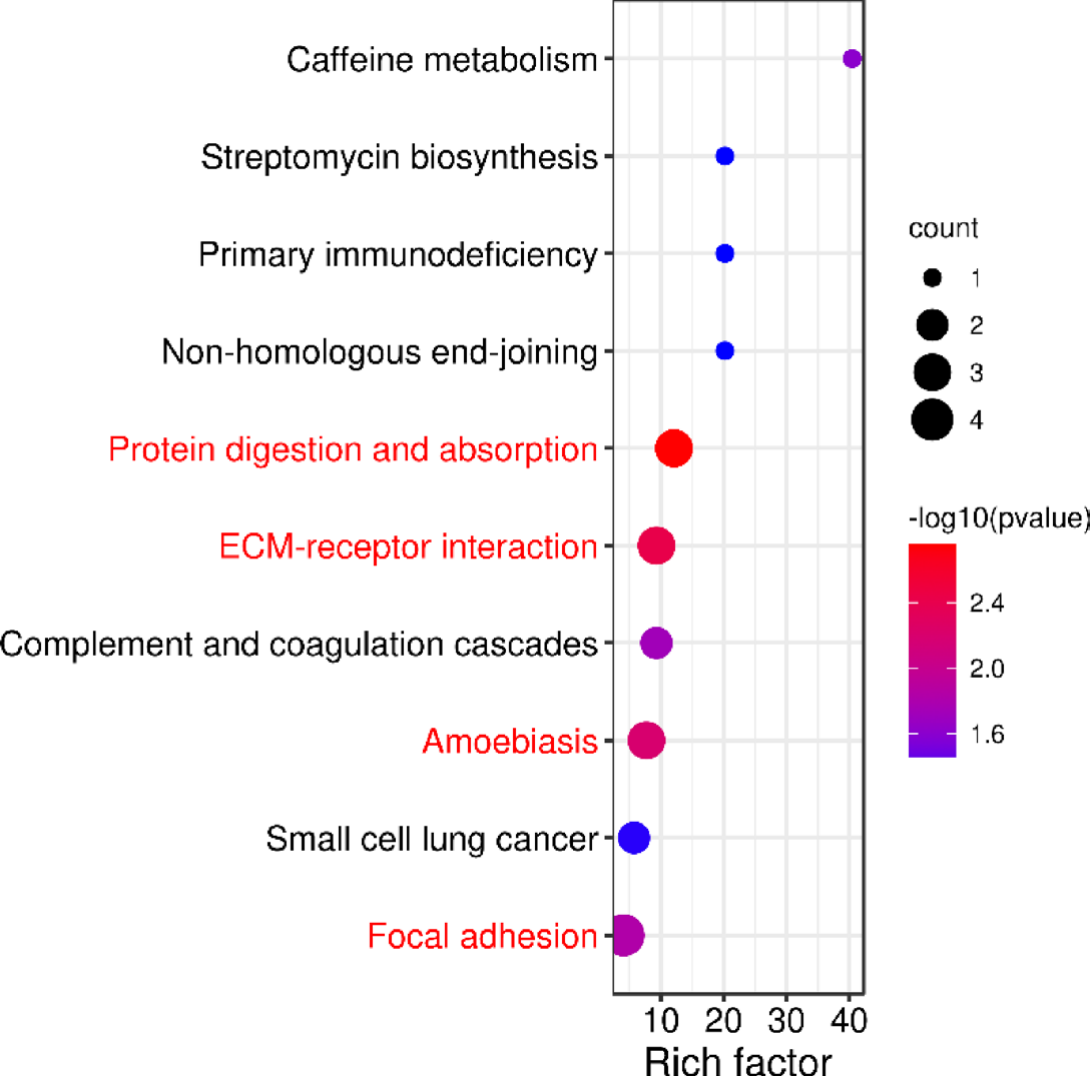
KEGG pathway enrichment of DEPs. The X-axis represents the enrichment factor (rich factor), which indicates the number of DEPs annotated to each pathway divided by all identified proteins annotated to the same pathway. The color of the bubble reflects the -log10-transformed P value, and the size of the bubble indicates the number of proteins enriched in the relevant pathway. Terms referenced in the text are marked in red.

The KEGG enrichment pathways of the DEPs were obtained via the KEGG database, and the criterion for significant enrichment was *p* < 0.05. Of the 69 differentially expressed proteins, 29 were annotated to the KEGG pathway, including 19 upregulated and 10 downregulated proteins. Among them, ten proteins were significantly enriched in the KEGG pathway, including seven upregulated and three downregulated proteins (Fig. 8). The focal adhesion pathway was associated with the highest number of proteins, followed by the protein digestion and absorption pathway, the ECM-receptor interaction pathway, and the amoebiasis-related pathway. Proteins in each pathway can be accessed in the Supplementary Table 5.

### PPI network analysis revealed that Itgb2 was the primary DEP

**Fig. 9:**
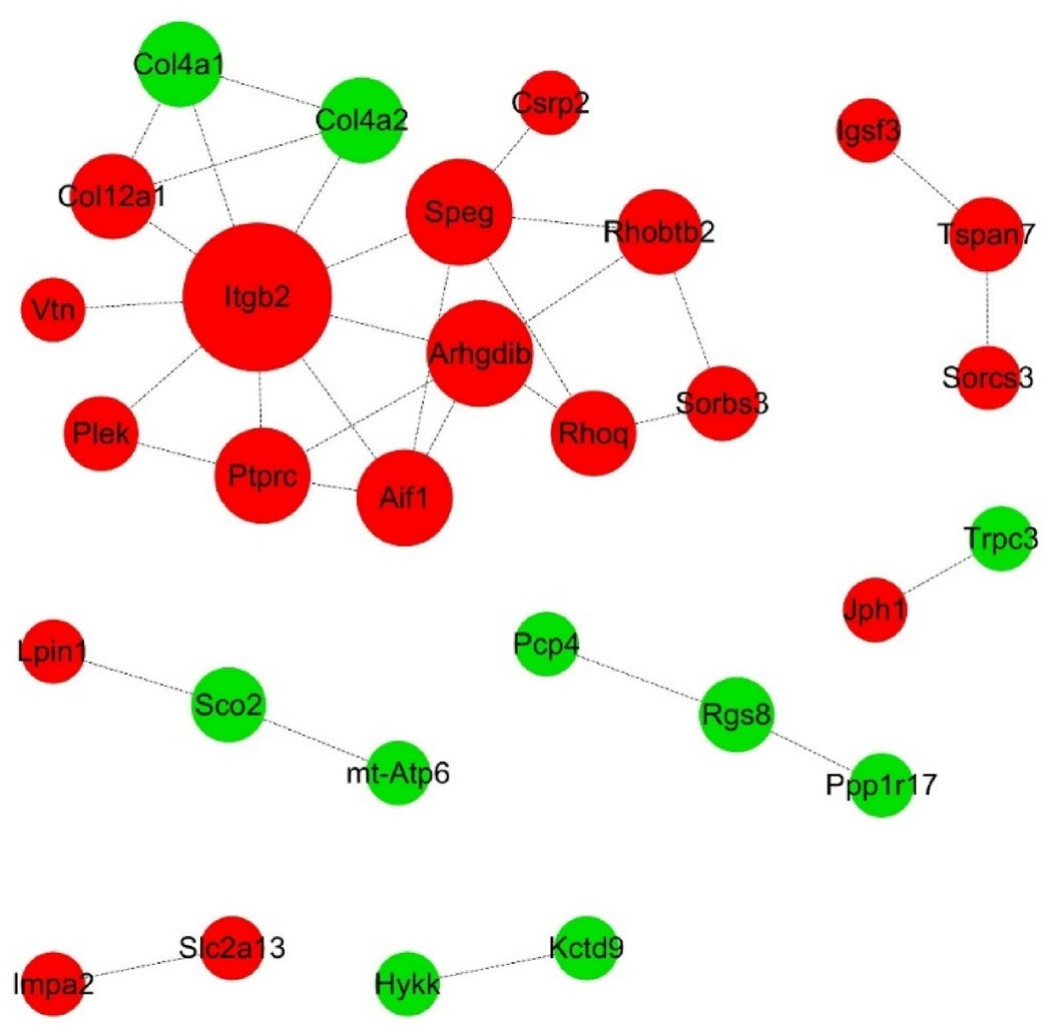
PPI network analysis of 29 DEPs. Red and green circles represent upregulated and downregulated proteins, respectively. The size of the circles indicates the node degree; the larger the circle is, the greater the node degree.

To better understand the pathogenic mechanisms of abnormal Ankfy1 expression in the cerebellum, a PPI network analysis of 69 altered proteins was performed with STRING and Cytoscape software (Fig. 9). STRING PPI network analysis revealed that 29 of the 69 DEPs interacted, with an average node degree of 0.957, an average local clustering coefficient of 0.289, and a PPI enrichment p-value of less than 8.36e-05. As shown in Fig. 9, integrin beta-2 (ITGB2) had the highest node degree and was at the core of the PPI network, followed by receptor-type tyrosine-protein phosphatase C (PTPRC), Rho GDP-dissociation inhibitor 2 (ARHGDIB), and allograft inflammatory factor 1 (AIF1).

### Validation of differential proteins

**Fig. 10:**
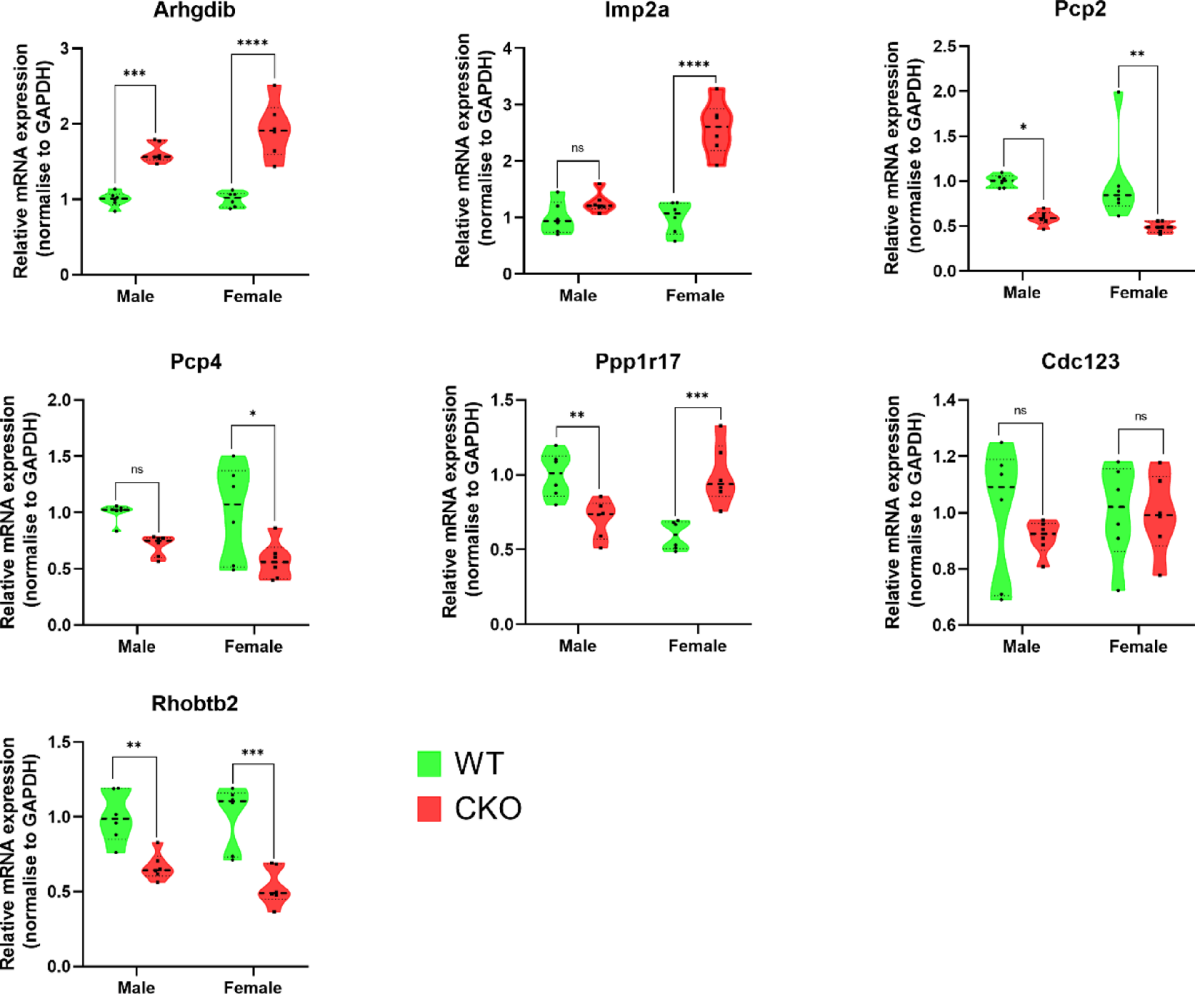
RT‒qPCR analysis of differentially expressed proteins. Violin plots of RT‒qPCR data from female (n: WT = 6, CKO = 6) and male mouse (n: WT = 6, CKO = 6) cerebellums (green represents the WT group, and red represents the CKO group) showing the transcript levels of the *Arhgdib*, *Impa2*, *Pcp2*, *Pcp4*, *Ppp1r17*, *Cdc123* and *Rhobtb2* genes between the CKO group and the WT group. Gene expression was normalized to GAPDH levels, and the data are shown as the mean fold change relative to that of WT mice (*p < 0.05, **p < 0.01, ***p < 0.001, ****p < 0.0001).

We selected up- and down-regulated differentially expressed proteins for RT‒qPCR validation (Fig. 10). These selected differentially expressed proteins had smaller P values and more prominent fold changes. According to the DIA analysis, inositol monophosphatase 2 (IMPA2), ARHGDIB, and Rho-related BTB domain-containing protein 2 (RHOBTB2) were increased. In contrast, the expression of Purkinje cell protein 2 (PCP2), calmodulin regulator protein PCP4 (PCP4), protein phosphatase one regulatory subunit 17 (PPP1R17), and cell division cycle protein 123 (CDC123) decreased. After RT-qPCR verification, it was confirmed that the expressions of *Arhgdib* and *Imp2a* were elevated, while those of *Pcp2* and *Pcp4* were decreased, which was consistent with the results of DIA analysis (Fig. 10). The expression of *Cdc123* RNA was lower in the CKO group than in the WT group, but the difference was insignificant (Fig. 10). The RNA expression of *Rhobtb2* that functions in the endocytosis is opposite to the result of DIA. Whether or not it is affected by ANKFY1 during the process of endocytosis still needs further verification. Interestingly, our RT-qPCR results show that the expression of ppp1r7 is opposite in female and male CKO mice (Fig. 10). Studies have confirmed that *Ppp1r17* can slow down the cell cycle progression of neural progenitor cells and is a putative gene for accelerating regional regulation^14^. Whether the expression differences of *Ppr1r17* in different genders play a unique role in neurodevelopment still requires further research to clarify.

**Fig. 11:**
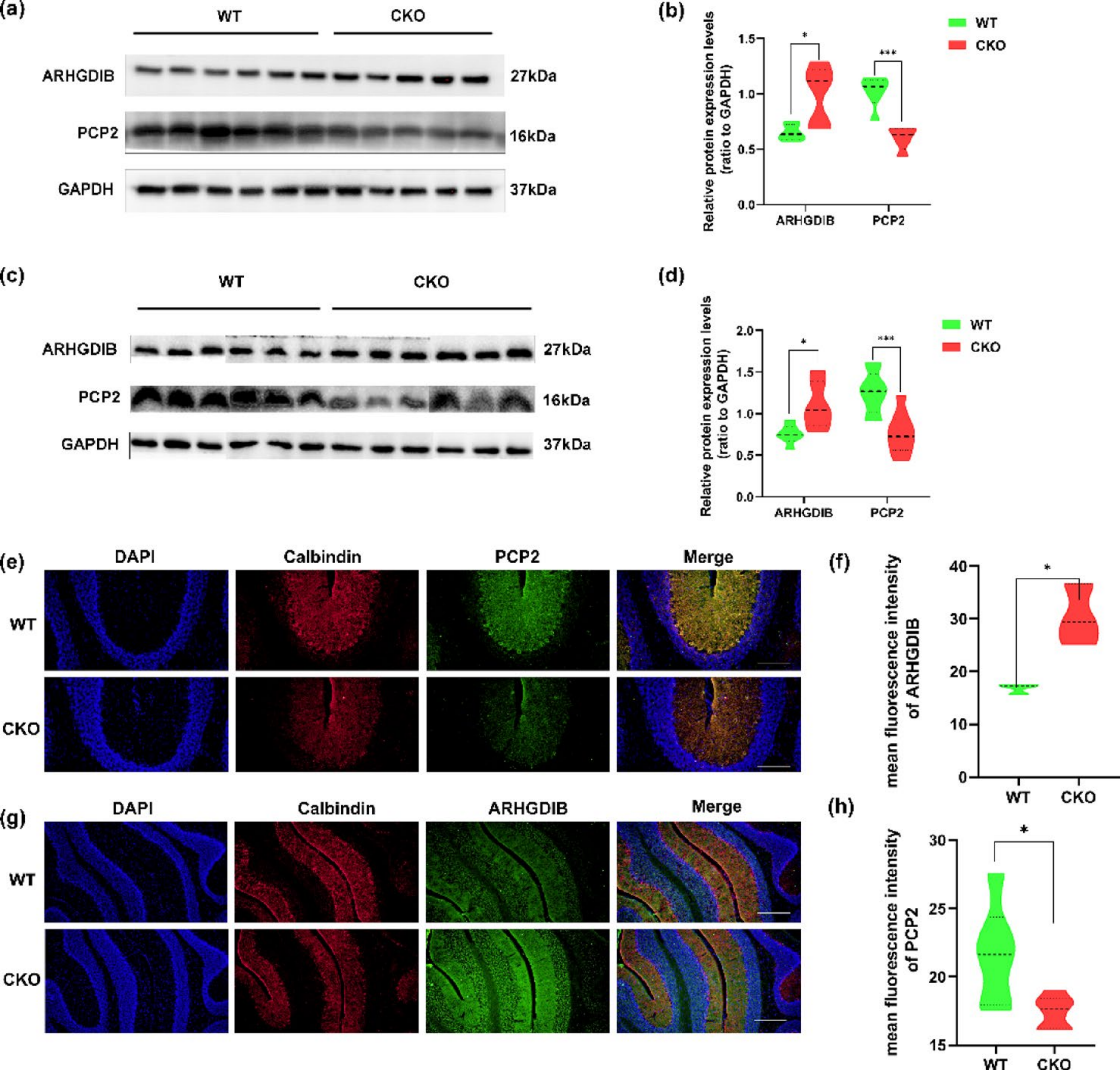
Immunity level validation of ARHGDIB and PCP2. (**a**) and (**c**) ARHGDIB and PCP2 expression in male (n: WT = 6, CKO = 5) and female mice (n: WT = 6, CKO = 6), respectively. Both studies demonstrated that ARHGDIB was elevated and PCP2 was decreased in the CKO group compared to the WT group in male and female mice, whereas GAPDH was used as a loading control. (**b**) and (**d**) Quantitative analysis showing a significant increase in the ARHGDIB level and a decrease in the PCP2 level in male and female mice, respectively (**b**, ARHGDIB: *t (9)* = 2.97; *P* = 0.016; PCP2: *t (9)* = 5.419; *P* = 0.0004; **d**, ARHGDIB: *t (10)* = 2.803;* P* = 0.0187; Pcp2: *t (16)* = 4.169; *P* = 0.0007) (*p < 0.05, ***p < 0.001). (**e**) and (**g**) Immunofluorescence staining images of the 4th and 5th cerebellar lobules from 7-month-old WT and CKO male mice are presented. Images of immunofluorescence staining (n = 3, 20× magnification in e and 10× magnification in g). Calbindin staining was used to reveal Purkinje cells. The scale bar represents 100 µm in image (**e**) and 200 µm in image (**g**). (**f**) and (**h**) Quantitative analysis of the mean fluorescence intensity of ARHGDIB throughout the entire cerebellar layer and PCP2 in the molecular layer and Purkinje cell layer of the cerebellum of 4th and 5th cerebellar lobules in WT and CKO mice (ARHGDIB: *t* (4) = 4.004; *P* = 0.0161; PCP2:* t* 10) = 2.647; *P* = 0.0244) (∗ p < 0.05, ** p < 0.01). The data are presented as the means ± SEMs.

Proteomic data suggested that ARHGDIB was near the center of the PPI network. Because of the specific expression of PCP2 in Purkinje cells, we postulated that ARHGDIB and PCP2 are the critical target proteins related to ANKFY1 deficiency-induced spastic ataxia. To further verify the expression levels of ARHGDIB and PCP2 in cerebellar tissues, Western blot analysis was used to evaluate the expression of these two proteins in female and male mice. These results were consistent with the RT‒qPCR results, with a significant decrease in PCP2 and an apparent increase in ARHGDIB (Fig. 11). Immunofluorescence staining of the cerebellum of normal and CKO mice revealed that the CKO mice exhibited a significant loss of Purkinje cells, accompanied by a reduced intensity of PCP2 in the molecular layer and Purkinje cell layer and an increase in ARHGDIB in the whole cerebellar layer of the 4th and 5th cerebellar lobules (Fig. 11e–f).

## Discussion

Recessive spastic ataxia is a combination of cerebellar ataxia and spastic paraplegia. A few inherited ataxias can manifest with a predominant spastic ataxia phenotype^1,2^. Our previous study demonstrated that *Ankfy1* knockout mice (*Ankfy1/*+) develop spastic paraplegia and ataxia with Purkinje cell loss in the mouse cerebellum^9^. Still, the underlying molecular mechanisms involved remain to be explored. This study established a mouse model of conditional *Ankfy1* knockout in cerebellar Purkinje cells. By investigating the impaired expression of Ankfy1 in Purkinje cells, we aimed to identify potential protein molecules involved in recessive spastic ataxia that may be targeted for disease diagnosis or treatment. Behavior deficits occurred at six months in the rotarod test, while Purkinje cell loss was observed in seven-month-old CKO mice (Fig. 10). We investigated the DEPs between WT and CKO mice through DIA-based proteomics and bioinformatics analyses. With 7224 proteins identified, 69 differentially expressed proteins were found, including 49 upregulated and 24 downregulated proteins.

According to the BP GO enrichment analysis, two upregulated proteins, AIF1 and ITGB2, were enriched for glial cell activation entry. Glial cells play an essential role in the CNS by influencing neuronal migration, axon specification and growth, coordinating circuit-wide neuronal differentiation, and regulating synapse formation and pruning^15^. There are no studies on whether these two proteins are associated with ataxia. Our previous study revealed that *Ankfy1/*+ mice exhibited increased expression of GFAP in the cerebellum, substantia nigra, and vestibular nuclei^9^, which affects most of the signals involved in functional alterations and morphology observed during astrocyte activation. These results indicate that reactive astrocytes and gliosis may be involved in ANKFY1 deficiency in mice.

Dynein light chain Tctex-type 1 (DYNLT1), which is associated with apical cargo transport, binding to transport cargo, and intracellular retrograde motility of vesicles and organelles along microtubules^16^, was upregulated in our study. GO analysis of the CC data indicated that three upregulated proteins, ITGB2, Cochlin (Coch), and Rho-related GTP-binding protein RhoQ (RHOQ), in the BP category were enriched in the regulation of cell morphology. PPI network analysis demonstrated that ITGB2 was at the network’s core and connected with substances in the extracellular matrix, such as vitronectin (VTN) and collagen (COL4A1 and COL4A2) (Fig. 10). Collagens are the most abundant proteins in mammals; they can interact with cells through several receptors, such as integrins, and play essential roles in regulating cell growth, differentiation, and migration^17^. Our mass spectrometry data demonstrated that the levels of COL4A1 and COL4A2 were apparently lower in the CKO group than in the WT group. The *Col4a1* and *Col4a2* genes encode collagen IV mutations that may cause various clinical disorders, including ocular, cerebral, renal, and muscular defects^18^. A multisystem syndrome called HANAC, which indicates hereditary angiopathy with nephropathy, aneurysms, and muscle cramps or elevated serum creatine kinase, is caused by mutations in *Col4a1*^18^, 2007, and Plaisier et al.^19^. Moreover, *Col4a2* mutations phenocopy *Col4a1* mutations and contribute to equally diverse disorders^18^. Neuronal cell morphogenesis depends on the proper regulation of microtubule-based transport; numerous examples of extensive rearrangements of the microtubule cytoskeleton have been described during morphogenesis by serving as a target in mitosis and providing the core structure, the mitotic spindle, to allow cell division to occur^20–22^. It has been reported that ANKFY1 is essential for the localization of integrin β1 on the cell surface^23^, so microtubule defects and dysfunction are considered possible causes of the pathogenesis of *Ankfy1* CKO mice.

In addition to collagen, other downregulated proteins, including the calmodulin regulator protein PCP4, protein phosphatase 1 regulatory subunit 17 (PPP1R17), and regulator of G-protein signaling 8 (RGS8), were found to participate in the regulation of the calcium signaling pathway. PPP1R17 is highly expressed in the cerebellum, and the other two genes, *GSBS* and *C7orf16*, which are G-substrate genes in humans, play a role in NO-sGC-cGMP-PKG pathway cerebellum-dependent long-term memory and neuroprotection of the ventral tegmentum and retina^24^, also known as PEP-19, is a small IQ motif-containing protein of the calpacitin family of proteins and a negative regulator of calmodulin and Ca^2+^/calmodulin-dependent kinase II-dependent (CaMKII-dependent) signaling in neurons^25^. A recent study showed that PCP4 contributes to the pathogenesis of AD by affecting Aβ protein processing and may be a novel therapeutic target for Alzheimer’s disease^26^. RGS8 regulates G protein-coupled receptor signaling cascades by increasing the GTPase activity of G protein alpha subunits; it inhibits signal transduction, thereby driving them into their inactive GDP-bound form^27^. It is specifically expressed in mouse cerebellar Purkinje cells; increasing its expression may mediate mGluR1 pathway dysregulation in Purkinje cells^28^.

The deletion of *Ankfy1* in mouse Purkinje cells may cause a significant decrease in the expression of mitochondrial ATP synthase six (*Mtatp6*) and the protein SCO2 homolog Sco2, both of which are crucial regulators of mitochondrial function. Pathogenic *Mtatp6* variants result in diverse biochemical features, including a reduced ATP synthesis rate, preserved ATP hydrolysis capacity, and abnormally increased mitochondrial membrane potential^29^. Researchers have noted that the most frequent symptoms caused by *Mtatp6* mutations are ataxia (81%), cognitive dysfunction (49%), neuropathy (48%), seizures (37%), and retinopathy (14%)^30^. Its mutations may also lead to adult-onset spinocerebellar ataxia^31^. Cytochrome c oxidase (COX) assembly protein SCO2 is a mitochondrial copper binding protein necessary for the assembly of COX complex IV of the mitochondrial respiratory chain; its mutation causes early-onset axonal Charcot-Marie-Tooth disease and cerebellar ataxia^32,33^. The PPI network showed that Sco2 was connected to Mtatp6. In addition, another Sco2 connection protein, phosphatidate phosphatase LPIN1, was upregulated according to the PPI network analysis. Lpin1 acts as a phosphatidate phosphatase enzyme required for glycerolipid biosynthesis and as a transcriptional coactivator that regulates lipid metabolism^34^. Its variants are associated with myopathy^35^ and severe rhabdomyolysis^36^. These results suggest that abnormalities in mitochondrial function and lipid metabolism may also regulate cerebellar function in Ankfy1-deficient mice.

PCP2, called Purkinje cell protein 2, is a GPR family member that interacts with the Gαi/o family of G proteins^37^. It is specifically and abundantly expressed in cerebellar Purkinje cells and retinal bipolar neurons. Previous studies have shown that PCP2 plays an essential role in the differentiation of cerebellar Purkinje cells; it can stimulate neurite formation and NGF-stimulated neurite growth through the activation of the Ras and p38 MAPK pathways^38,39^. Previous studies have found that Purkinje cell loss occurs in *Ankfy1/*+ mice^9,10^. Our study confirms that *Ankfy1* CKO mice also show a loss of Purkinje cells (Fig. 10). We hypothesized that reduced Pcp2 expression is associated with *Ankfy1*-induced deletion of Purkinje cells.

Rho-GTPases are a branch of the Ras-related small GTP-binding protein superfamily. They are signaling molecules involved in many cellular processes, such as cell growth, cytoskeleton organization, and secretion^40^. Rho binds to GDP to form an inactivated state, while GTP binds to an activated state. A GDP-dissociation inhibitor (GDI) blocks Rho activation by binding to inactive Rho-GDP, thereby decreasing the dissociation rate^41^. RhoA signaling is important in neurodegenerative diseases, such as Parkinson’s disease, Alzheimer’s disease, and Huntington’s disease, which are characterized by increased RhoA and ROCK expression, and by their inhibition, these diseases can rescue lesions^42^. ARHGDIB, Rho GDP-dissociation inhibitor 2, RhoGDI2 or Ly-GDI, is a GDP-GDI of the RhoA GTP-binding protein and is expressed in many tissues. Studies have demonstrated that it can inhibit the growth and metastasis of bladder cancer^41,43,44^. Our study also revealed increased GDI expression in the cerebellum of Ankfy1 CKO mice. PPI network analysis of the differentially expressed proteins revealed that the expression of Rho-related proteins such as RHOBTB2 and RHOQ was upregulated. It was hypothesized that the RhoA signaling pathway may be involved in Ankfy1 deficiency in mice, and that the increase in GDI may be a negative feedback loop resulting from overactivation of the RhoA signaling pathway. Previous research has also demonstrated that RhoD GTPase activating protein and VPS9 domain 1 (GAPVD1) can bind to Ankfy1 and collaboratively regulate RAB5-mediated endocytic trafficking^6,45^. We also hypothesized that Rho-GTPase might be involved in spastic ataxia caused by ANKFY1 deficiency.

## Limitations

To minimize biological variation due to sex differences, we randomly selected male mice for the subsequent mass spectrometry analysis. To address potential sex-related biases, we validated key findings using RT‒qPCR and Western blotting in both sexes, and the results were consistent between male and female mice. In the future, gender-specific proteomic profiles may be systematically analyzed in expanded cohorts.

In addition, for differential protein validation, we focused on seven-month-old male mice and prioritized 7 of the 69 differentially expressed proteins for further verification. In the future, additional differentially expressed proteins warrant validation, the temporal dynamics of these protein changes, whether they occur earlier than seven months, remain to be determined, and the pathogenic mechanisms of the relevant differentially expressed proteins must be explored in greater depth.

## Conclusion

This study employed the DIA-based proteomics method to compare and analyze DEPs between wild-type mice and conditional Ankfy1 knockout mice. Among the 69 DEPs, the expression of some *Ankfy1*-related proteins was proven to be involved in neurodegenerative disease. However, many other DEPs have not yet been identified in the cerebellar tissue of patients with spastic ataxia, which provides reference proteins for further research.

## Electronic supplementary material

Below is the link to the electronic supplementary material.

Supplementary Material 1

Supplementary Material 2

Supplementary Material 3

## Data Availability

The data are available upon request from the corresponding authors. Proteomics data supporting this study’s findings have been deposited in PRIDE with the project accession: PXD057098, Token: PGJ4cT2oGdJ. (https://www.ebi.ac.uk/pride/). Uniport database (https://www.uniprot.org/), KEGG database (https://www.genome.jp/kegg/).

## Abbreviations

ANKFY1: Ankyrin repeat and FYVE domain containing 1
DIA: Data-independent acquisition
MS: Mass spectrometry
CKO: Conditional knockout
WT: Wild-type
DEP: Differentially expressed protein
CNS: Central nervous system
ARSACS: Autosomal recessive ataxia of Charlevoix-Saguenay
GO: Gene Ontology
DMEM: Dulbecco’s modification of Eagle’s medium
COG: Orthologous Groups of Proteins
KEGG: Kyoto Encyclopedia of Genes and Genomes
PCP2: Purkinje cell protein 2
PPP1R17: Protein phosphatase 1 regulatory subunit 17
RGS8: Regulator of G-protein signaling 8
MTATP6: Mitochondrial ATP synthase six
GDI: GDP-dissociation inhibitor
PTPRC: Receptor-type tyrosine-protein phosphatase C
ARHGDIB: Rho GDP-dissociation inhibitor 2
AIF1: Allograft inflammatory factor 1
IMPA2: Inositol monophosphatase 2
RHOBTB2: Rho-related BTB domain-containing protein 2
CDC123: Cell division cycle protein 123
DYNLT1: Dynein light chain Tctex-type 1
PCR: Polymerase chain reaction
RT-QPCR: Real-time quantitative PCR
shRNA: Short hairpin RNA
GAPDH: Glyceraldehyde 3-phosphate dehydrogenase
FBS: Fetal bovine serum
PBS: Phosphate-buffered saline
RIPA: Protease inhibitor-containing radioimmunoprecipitation
BSA: Bovine serum albumin
SDC: Sodium dodecyl sulfate
DAPI: 4′,6-Diamidino-2-phenylindole
FDR: False discovery rate
NOG: Nonsupervised Orthologous Groups
BP: Biological processes
CC: Cellular components
MF: Molecular functions
PPI: Protein–protein interaction
PCA: Principal component analysis
ANOVA: Analysis of variance
ECM: Extracellular matrix
