# Supplementary material for: Identification of spastic ataxia-related proteins via comparative proteomic analysis of the cerebellum of conditional Ankfy1 knockout mice
Source: Sci Rep. 2025 Jul 1;15:20683. doi: 10.1038/s41598-025-06398-8 (PMC12217688; doi:10.1038/s41598-025-06398-8)
Supplement: Supplementary file 1 — Supplementary Material 1 [file 41598_2025_6398_MOESM1_ESM.pdf]

Supplemental Tables

Table 1. List of up-regulated proteins.

Accession	Gene	Description	CKO/WT	P-value
Q3UIZ8	Mylk3	Myosin light chain kinase 3	57.80486	8.66E-05
Q91UZ5	Impa2	Inositol monophosphatase 2	4.810771	0.003948
Q9ES07	Slc15a2	Solute carrier family 15 member 2	3.964321	0.014371
Q8BP56	Pgghg	Protein-glucosylgalactosylhydroxylysine glucosidase	3.77239	0.02039
Q61599	Arhgdib	Rho GDP-dissociation inhibitor 2	3.628868	0.008689
Q9R1Z8	Sorbs3	Vinexin	3.304832	0.017986
P29788	Vtn	Vitronectin	3.040589	0.01247
P51807	Dynlt1	Dynein light chain Tctex-type 1	2.967796	0.04969
O70200	Aif1	Allograft inflammatory factor 1	2.840195	0.006335
Q3UBG2	Pid1	PTB-containing, cubilin and LRP1-interacting protein	2.732563	0.022745
Q8BT14	Cnot4	CCR4-NOT transcription complex subunit 4	2.664637	0.026422
Q60673	Ptprn	Receptor-type tyrosine-protein phosphatase-like N	2.563901	0.044515
Q8R527	Rhoq	Rho-related GTP-binding protein RhoQ	2.508277	0.036677
Q99NF2	Nsmf	NMDA receptor synaptonuclear signaling and neuronal migration factor OS=Mus musculus	2.502062	0.011569
Q9JHK5	Plek	Pleckstrin OS=Mus musculus	2.372021	0.007045
A3KGF9	Ccdc9b	Coiled-coil domain-containing protein 9B	2.321924	0.042091
Q8CAS9	Parp9	Protein mono-ADP-ribosyltransferase PARP9	2.151325	0.031527
P97304	Polr1d	DNA-directed RNA polymerases I and III subunit RPAC2	2.05982	0.020429
Q62507	Coch	Cochlin	2.017222	0.035712
E9Q4S1	Pde8b	High affinity cAMP-specific and IBMX-insensitive 3',5'-cyclic phosphodiesterase 8B	1.980631	0.009312
P11835	Itgb2	Integrin beta-2	1.970762	0.018799
Q9WTZ1	Rnf7	RING-box protein 2	1.958339	0.036492
Q60847	Col12a1	Collagen alpha-1(XII) chain	1.925099	0.001984
P06800	Ptprc	Receptor-type tyrosine-protein phosphatase C	1.916854	0.04026
Q8VI51	Sorcs3	VPS10 domain-containing receptor SorCS3	1.859159	0.036102
Q9DC22	Dcaf6	DDB1- and CUL4-associated factor 6	1.85846	0.008857
O09114	Ptgds	Prostaglandin-H2 D-isomerase	1.842974	0.021408
O54818	Tpd52l1	Tumor protein D53	1.770084	0.047838
Q3UHK1	Slc2a13	Proton myo-inositol cotransporter	1.769993	0.034349
Q9ET80	Jph1	Junctophilin-1	1.762521	0.01097
Q6ZQA6	Igsf3	Immunoglobulin superfamily member 3	1.757356	0.021955
P97314	Csrp2	Cysteine and glycine-rich protein 2	1.744712	0.025715
P97799	Nrsn1	Neurensin-1	1.735835	0.009293
Q60632	Nr2f1	COUP transcription factor 1	1.73356	0.04655
Q8BU27	Ppm1m	Protein phosphatase 1M	1.728654	0.030521

Q91V93	Rhobtb2	Rho-related BTB domain-containing protein 2	1.69514	0.040951
Q62283	Tspan7	Tetraspanin-7	1.674376	0.041731
Q91ZP3	Lpin1	Phosphatidate phosphatase LPIN1	1.626831	0.018765
Q8BFW4	Trim65	Tripartite motif-containing protein 65	1.583778	0.012859
Q62407	Speg	Striated muscle-specific serine/threonine-protein kinase	1.570887	0.015417
Q80U04	Pja2	E3 ubiquitin-protein ligase Praja-2	1.565099	0.010493
Q80T69	Rsbn1	Lysine-specific demethylase 9	1.558382	0.040717
A2RTL5	Rsrc2	Arginine/serine-rich coiled-coil protein 2	1.556011	0.042689
Q8VCM3	Zfyve21	Zinc finger FYVE domain-containing protein 21	1.505002	0.037585
P27641	Xrcc5	X-ray repair cross-complementing protein 5	1.5012	0.033678

Table 2. List of down-regulated proteins.

Accession	Gene	Description	CKO/WT	P-value
B9EKX1	Ptchd4	Patched domain-containing protein 4	0.657202	0.012645
O54949	Nlk	Serine/threonine-protein kinase NLK	0.618268	0.039461
P00848	Mtatp6	ATP synthase subunit	0.658977	0.037111
P02463	Col4a1	Collagen alpha-1(IV) chain	0.494113	0.000722
P08122	Col4a2	Collagen alpha-2(IV) chain	0.479401	0.003038
P12660	Pcp2	Purkinje cell protein 2	0.64789	0.028429
P61804	Dad1	Dolichyl-diphosphooligosaccharide--protein glycosyltransferase subunit DAD1	0.634398	0.048016
P63054	Pcp4	Calmodulin regulator protein PCP4	0.649514	0.006938
Q3U1T9	Dennd1b	DENN domain-containing protein 1B	0.559103	0.03318
Q5U5V2	Hykk	Hydroxylysine kinase	0.649307	0.007713
Q80UN1	Kctd9	BTB/POZ domain-containing protein KCTD9	0.593131	0.018701
Q80WR5	C1orf174	UPF0688 protein C1orf174 homolog	0.587071	0.008548
Q8BG17	Nol12	Nucleolar protein 12	0.460558	0.0291
Q8BGZ2	Fam168a	Protein FAM168A	0.585429	0.016344
Q8BXT1	Rgs8	Regulator of G-protein signaling 8	0.655942	0.031853
Q8VCL2	Sco2	Protein SCO2 homolog, mitochondrial	0.65375	0.023836
Q9D142	Nudt14	Uridine diphosphate glucose pyrophosphatase	0.59115	0.01384
Q9QZC1	Trpc3	Short transient receptor potential channel 3	0.638449	0.046244
Q9Z2E4	Ppp1r17	Protein phosphatase 1 regulatory subunit 17	0.415711	0.034515
Q0VG49	C15orf61	Uncharacterized protein C15orf61 homolog	0.309394	0.029448
Q62036	Cep131	Centrosomal protein of 131 kDa	0.142946	0.036983
P15392	Cyp2a4	Cytochrome P450 2A4	0.399556	0.000265
Q8CII2	Cdc123	Cell division cycle protein 123 homolog	0.304733	0.029161
Q9ER47	Kcnh7	Potassium voltage-gated channel subfamily H member 7	0.410643	0.03685
