# Supplementary material for: Identification of spastic ataxia-related proteins via comparative proteomic analysis of the cerebellum of conditional Ankfy1 knockout mice
Source: Sci Rep. 2025 Jul 1;15:20683. doi: 10.1038/s41598-025-06398-8 (PMC12217688; doi:10.1038/s41598-025-06398-8)
Supplement: Supplementary file 2 — Supplementary Material 2 [file 41598_2025_6398_MOESM2_ESM.pdf]

Table 3. GO enrichment gene

GO	Name	p-value	Count	Accession-up	Accession-down
BP	formation of primary germ layer	0.000443	4	P11835; P29788; Q60847	P08122
	leukocyte activation involved in inflammatory response	0.00367	2	O70200; P11835	
	phototransduction	0.00446	2		P12660; Q9QZC1
	glial cell activation	0.00625	2	O70200; P11835	
	postsynaptic signal transduction	0.00625	2	Q99NF2	Q8BXT1
	regulation of response to stimulus	0.00729	24	O54818; O70200; P06800; P11835; P27641; P29788; P51807; Q3UBG2; Q60632; Q61599; Q62507; Q80U04; Q8CAS9; Q8R527; Q91V93; Q9ET80; Q9JHK5; Q9R1Z8	O54949; P63054; Q3U1T9; Q8BGZ2; Q8BXT1; Q9QZC1
	phenylpropanoid metabolic process	0.00933	1		P15392
	manchette assembly	0.00933	1		Q62036
	intramanchette transport	0.00933	1		Q62036
	type 2 immune response	0.0186	1		Q3U1T9
	secretory granule maturation	0.0186	1	Q60673	
	protein secretion by platelet	0.0186	1	Q9JHK5	
CC	network-forming collagen trimer	0.0000856	2		P02463; P08122
	basement membrane collagen trimer	0.000255	2		P02463; P08122

	complex of collagen trimers	0.000508	2		P02463; P08122
	collagen-containing extracellular matrix	0.00124	4	P29788; Q60847	P02463; P08122
	supramolecular polymer	0.0036	9	O70200; P51807; Q8R527; Q9ET80	P02463; P08122; P15392; P63054; Q8VCL2
	FACIT collagen trimer	0.00933	1	Q60847	
	cell wall	0.00933	1	Q8BP56	
	anchoring collagen complex	0.00933	1	Q60847	
	cytoplasmic microtubule	0.0296	2	P51807	P15392
	telomere cap complex	0.0723	1	P27641	
MF	extracellular matrix constituent conferring elasticity	0.00933	1		P02463
	protein-glucosylgalactosylhydroxylysine glucosidase activity	0.00933	1	Q8BP56	
	ADP-D-ribose binding	0.00933	1	Q8CAS9	
	STAT family protein binding	0.0277	1	Q8CAS9	
	heparin binding	0.0315	2	P06800; P29788	
	collagen binding	0.0355	2	P29788; Q62507	
	opsonin binding	0.0458	1	P11835	
	carbohydrate transmembrane transporter activity	0.0458	1	Q3UHK1	
	oxygen binding	0.0458	1		P15392

Table 4. COG enrichment gene

**Class**	**Name**	**Pvalue**	**Count**	**Accession-up**	**Accession-down**
Cellular processes and	Extracellular structures	0.00113	5	P29788; Q60847; Q62507	P02463; P08122

signaling					
	Signal transduction mechanisms	0.0653	19	E9Q4S1; O70200; P06800; P11835; P97314; Q3UIZ8; Q60673; Q61599; Q62407; Q6ZQA6; Q8BU27; Q99NF2; Q9JHK5; Q9R1Z8	O54949; P12660; P63054; Q3U1T9; Q8BXT1
	Cell motility	0.0917	1	P51807	
	Defense mechanisms	0.456	1	Q62507	
	Posttranslational modification, protein turnover, chaperones	0.562	6	O09114; P29788; Q80U04; Q8BFW4; Q9WTZ1	P61804
	Cell cycle control, cell division, chromosome partitioning	0.684	1		P61804
	Cytoskeleton	0.892	2	P97314	Q62036
	Intracellular trafficking, secretion, and vesicular transport	0.976	2	Q3UHK1; Q8VI51	
Information storage and processing	Replication, recombination and repair	0.262	2	P27641	Q9D142
	Chromatin structure and dynamics	0.645	1	Q8CAS9	
	Transcription	0.905	4		P97304; Q60632; Q8CAS9; Q91ZP3
	RNA processing and modification	0.954	1	Q8BT14	
Metabolism	Carbohydrate transport and metabolism	0.452	2	Q8BP56; Q91UZ5	

	Secondary metabolites biosynthesis, transport and catabolism	0.556	1		P15392
	Inorganic ion transport and metabolism	0.596	2		Q9ER47; Q9QZC1
	Amino acid transport and metabolism	0.748	1	Q9ES07	
	Energy production and conversion	0.775	2		P00848; Q8VCL2
Poorly characterized	Function unknown	0.0232	21	A2RTL5; A3KGF9; O54818; P97799; Q3UBG2; Q62283; Q80T69; Q8R527; Q8VCM3; Q91V93; Q9DC22; Q9ET80	B9EKX1; Q0VG49; Q5U5V2; Q80UN1; Q80WR5; Q8BG17; Q8BGZ2; Q8CII2; Q9Z2E4

Table 5. KEGG enrichment gene

**Pathway Name**	**Class**	**Pvalue**	**Count**
Focal adhesion	Cellular Processes	0.0149	4
ECM-receptor interaction	Environmental Information Processing	0.00377	3
Non-homologous end-joining	Genetic Information Processing	0.0485	1
Amoebiasis	Human Diseases	0.00641	3
Small cell lung cancer	Human Diseases	0.0461	2
Primary immunodeficiency	Human Diseases	0.0485	1
Caffeine metabolism	Metabolism	0.0245	1
Streptomycin biosynthesis	Metabolism	0.0485	1
Protein digestion and absorption	Organismal Systems	0.00176	3

Complement and coagulation cascades	Organismal Systems	0.0189	2
