# Supplementary material for: Identification of spastic ataxia-related proteins via comparative proteomic analysis of the cerebellum of conditional Ankfy1 knockout mice
Source: Sci Rep. 2025 Jul 1;15:20683. doi: 10.1038/s41598-025-06398-8 (PMC12217688; doi:10.1038/s41598-025-06398-8)
Supplement: Supplementary file 3 — Supplementary Material 3 [file 41598_2025_6398_MOESM3_ESM.pdf]

Supplemental Figures

Supplementary Figure S1

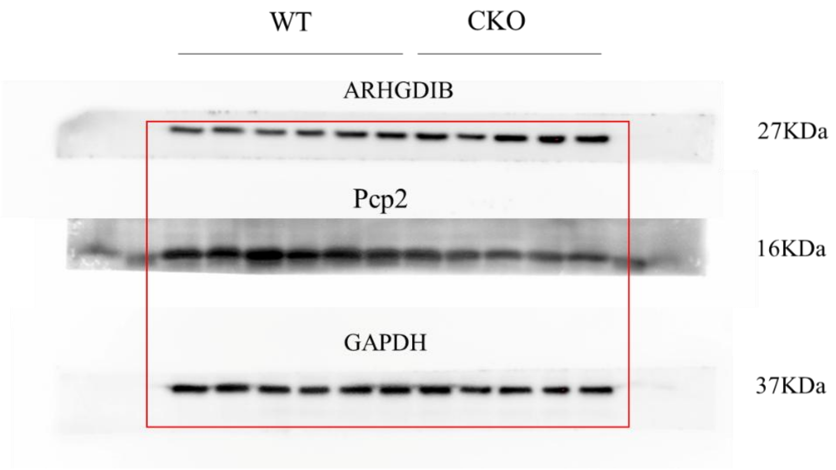

Supplementary Figure S1. Unprocessed original scans of Western blot analysis from Figure 11a. The red-dot-lined squared area is used to indicate the lanes and bands used in the Figures.

Supplementary Figure S2

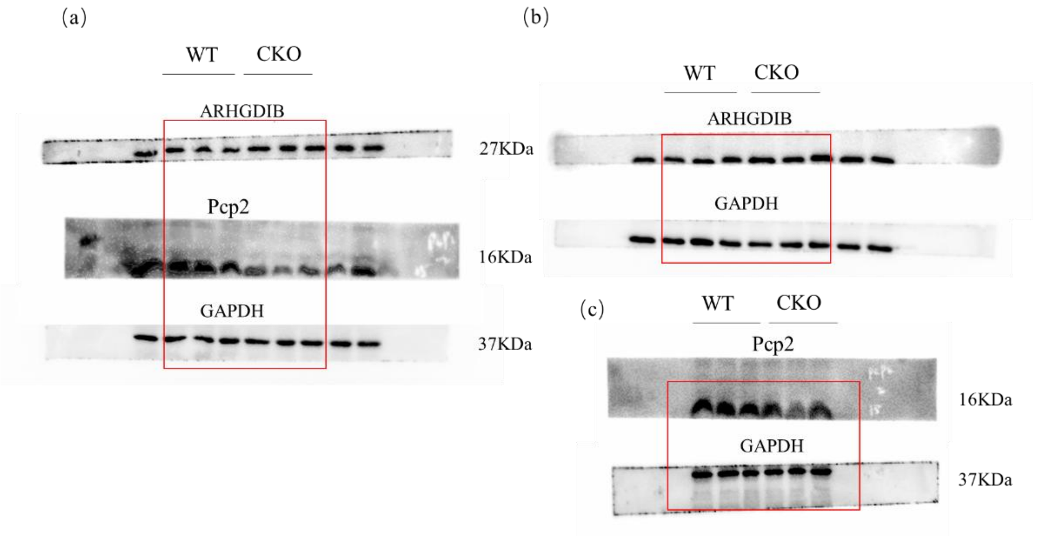

Supplementary Figure S2. Unprocessed original scans of Western blot analysis from Figure 11c. The red-dot-lined squared area is used to indicate the lanes and bands used in the Figures.

Supplementary Figure S3

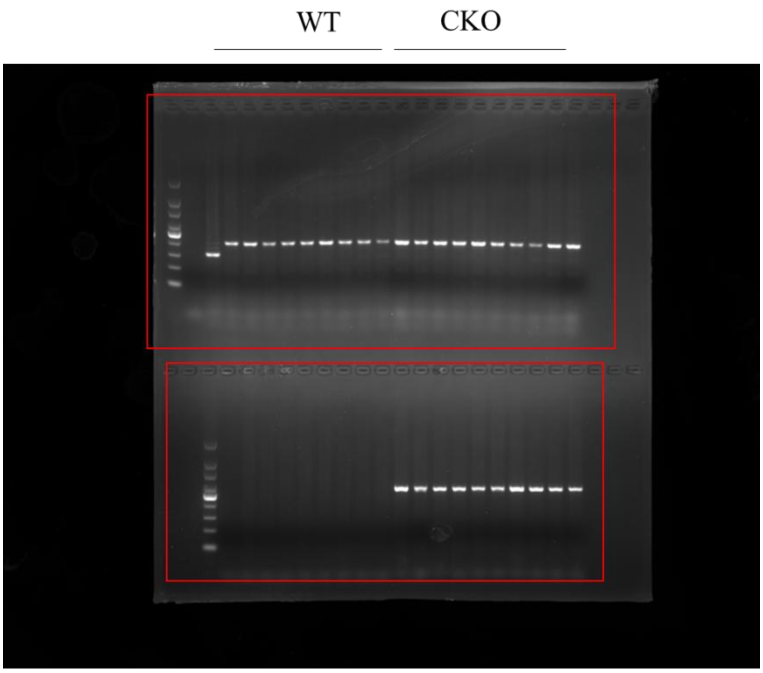

Supplementary Figure S3. Unprocessed original scans of agarose gel electrophoresis analysis from Figure 1b. The red-dot-lined squared area is used to indicate the lanes and bands used in the Figures.

Supplementary Figure S4

(a)

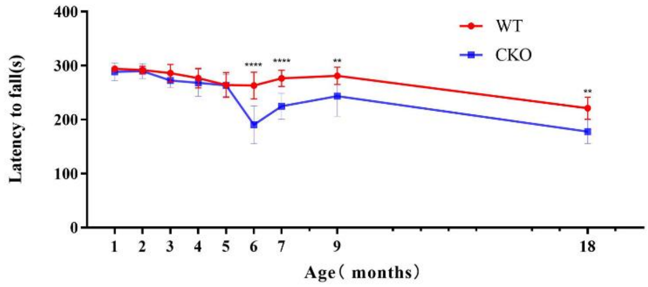

Supplementary Figure S4. The results of the rotarod test (to assess locomotor and coordination abilities) on mice aged 1 to 18 months (n = 6-12) showed that the motor function of CKO mice significantly declined starting from the 6th month. For 6-month-old mice, the time (in seconds)

when they fell off the rotarod was as follows: for WT group, it was 263.2 ± 23.7, and for the CKO group, it was 190.4 ± 33.6 (t = 5.776, P = 2.23464E-05) *P < 0.01, **P < 0.0001.
